# Caries experience and risk indicators of having decayed teeth among 65-year-olds in Oslo, Norway: a cross-sectional study

**DOI:** 10.1186/s12903-023-03432-x

**Published:** 2023-10-07

**Authors:** My Tien Diep, Rasa Skudutyte-Rysstad, Anne Thea Tveit Sødal, Alix Young, Lene Hystad Hove

**Affiliations:** 1 Department of Cariology and Gerodontology, Faculty of Dentistry, University of Oslo, P.O. Box 1109, Oslo, N-0317 Norway; 2 Oral Health Centre of Expertise in Eastern Norway, Sørkedalsveien 10A, Oslo, 0369 Norway

**Keywords:** Epidemiology, Caries prediction, Dental caries, Root caries, Caries experience, Risk indicators, Older adults

## Abstract

**Background:**

Meeting the oral health needs of the increasing population of older adults presents a major challenge in dental care. Knowledge about the oral health status in the young-elderly age group is essential for the planning of future oral health education and prevention programs. The aims of the present study were therefore to investigate the caries experience among 65-year-olds in Oslo, Norway, and to explore associations between having decayed teeth and sociodemographic, behavioural, and biological factors.

**Methods:**

A random sample of 65‑year‑olds in Oslo answered a questionnaire and underwent clinical and radiographic examinations (n = 457, 52% men and 48% women) at the Research Clinic, Faculty of Dentistry, University of Oslo, between February and December 2019. Primary- and secondary coronal and root caries lesions, root remnants, and missing and restored teeth were recorded. Decayed teeth (DT) were defined as teeth with coronal- and root caries lesions that had progressed into dentine and root remnants, and the DMFT/S scores were calculated.

**Results:**

The mean number of teeth was 25 (SD: 4) and the mean DMFT was 19.4 (SD: 4.7). Thirty seven percent of the individuals had at least one decayed tooth (DT > 0), and the mean number of filled teeth (FT) was 16.1 (SD: 5.4). Multivariable logistic regression analysis showed that male gender (OR: 1.8, 95% CI: 1.2–2.8), basic level of education (OR: 1.9, 95% CI: 1.2–2.9), irregular dental attendance (OR: 2.2, 95% CI: 1.0-4.8), and hyposalivation (OR: 2.1, 95% CI: 1.0-4.4) were significant risk indicators for having decayed teeth (DT > 0) (p < 0.05).

**Conclusions:**

In conclusion, 65-year-olds in Oslo had a low average number of decayed and missing teeth, and a high number of restored teeth. Irregular dental attendance and hyposalivation were the strongest risk indicators for having decayed teeth. Based on the present results, it will be important to ensure access to regular dental care and to increase the emphasis on caries preventive measures for individuals with hyposalivation in this age group.

**Supplementary Information:**

The online version contains supplementary material available at 10.1186/s12903-023-03432-x.

## Introduction

The post-World War II generation, known as the baby boomers [1], are now entering old age and represents the largest proportion of the older adult population in many countries. In Scandinavia, the majority of this cohort has nearly complete dentitions [2–4], and higher expectations of function and aesthetics, compared to previous cohorts [5]. These factors are likely to increase the demand for dental health services in the older adult population.

Old age is strongly associated with multimorbidity and increased medication use [6], both of which may increase the risk of dental caries due to oral dryness [7] and reduced ability to self-care [8]. In addition, age-related changes in the oral cavity, such as gingival recession and subsequent exposure of root surfaces, result in more tooth surfaces becoming susceptible to caries [9]. Along with periodontal disease, dental caries and its sequelae are one of the main causes of tooth loss among adults [10].

Dental caries is a multifactorial disease, determined by interactions between biological, behavioural, psychosocial, and environmental factors [11]. Inadequate oral hygiene [12], and frequent sugar intake [13, 14] are well known caries risk indicators. Furthermore, low income and/or lower level of education attainment are also associated with higher levels of untreated dental caries [15–18]. In addition, demographic variables, such as male gender [4, 19, 20] and country of origin [16], have also been reported to influence caries experience. The explanation may be that sociodemographic factors influence oral health behaviour, such as oral hygiene, dietary habits, and use of dental health services [18]. Moreover, financial hardship may be a reason for not attending routine dental check-ups [21], which in turn may influence caries control and management. The hierarchical arrangement of proximal (e.g. salivary status), intermediate (e.g. oral health-related behaviour), and distal (e.g. socio-demographic) effects should be taken into account in multivariable analyses [22].

Effect size of established caries risk indicators related to sociodemographic, behavioural, and biological factors, may vary depending on the population of interest. As fluoride toothpaste was first introduced in the early 1970’s, the current young-elderly population did not have access to fluoridated toothpaste until well after their permanent teeth had erupted [23]. Furthermore, when the current young-elderly were young adults, dentists had a more operative approach to treating dental caries [24]. In Norway, the criteria for restorative treatment of dental caries changed during the 1980s, from operative treatment of enamel caries lesions to delaying operative intervention until the lesion could be clearly registered radiographically in the dentine [25]. Limited use of fluoride toothpastes and the more invasive treatment approach may have contributed to more caries, dental restorations and tooth fractures in the current young-elderly population compared to younger generations. Therefore, caries risk indicators found in studies on younger age groups should also be explored in older age groups.

In Norway, dental health services are divided into a private and public sector [26, 27]. The majority of adults receive oral care from private general dental practitioners, mainly paid by the patients. Dental treatment provided by the Public Dental Health Service is free of charge for patients aged 0–18 years, mentally disabled adults, and older adults living in an institution or receiving home nursing care.

Age 65 years is defined as the beginning of old age [28, 29]. Currently, there are few studies describing caries experience in the young-elderly population, and epidemiological studies on the caries status of older adults, including assessment of behavioural and socioeconomic risk indicators, are much needed [30]. From a public health perspective, it is important to map caries experience in the young-elderly population for planning of future dental health services for this age cohort. In addition, knowledge about caries risk indicators in this age group is essential for the planning of oral health education and prevention programmes. The aims of the present study were therefore to investigate the caries experience among 65-year-olds in Oslo, Norway, and to explore the associations between having decayed teeth and sociodemographic, behavioural, and biological factors, accounting for hierarchical relationships between these variables.

## Materials and methods

### Study design and setting

The present cross-sectional study was part of a larger study investigating oral diseases and conditions in 65-year-olds in Oslo, Norway (the OM65 study). As previously described (31), the participants were examined at the Research Clinic, Faculty of Dentistry, University of Oslo, between February and December 2019. The Norwegian Regional Committee for Research Ethics approved the study protocol (REK 2018/1383), and the study was performed in compliance with the tenets of the Declaration of Helsinki. All participants signed a written informed consent form. The present paper was written using the ‘strengthening the reporting of observational studies in epidemiology’ (STROBE) guidelines [32].

### Participants

The inclusion criteria were being 65 years old (born in 1954), resident in Oslo, Norway, and being reachable by phone. The latter criteria was set for practical reasons in relation to booking of appointments for the examinations. There were no exclusion criteria. Assuming that the prevalences of the oral conditions of interest are 10% or more, and accounting for planned longitudinal follow-up studies with a potential total dropout rate of 70%, the calculated sample size was 450 participants. Eligible individuals were randomly selected from the Norwegian National Population Register, and invitation letters were sent out consecutively to these individuals until the calculated sample size was reached. At the end of the recruitment process we had sent out a total of 1230 invitation letters to these 65-year-olds. Within 2 weeks, all individuals were contacted by telephone, and individuals who answered were asked if they were interested in participating in the study.

### Questionnaire

Prior to attending the clinical examination at the Research Clinic, individuals who agreed to participate received an electronic link to a self-administered online questionnaire (Nettskjema®, University of Oslo). The participants were asked about their gender (male or female), country of birth, educational attainment, financial capacity related to affording dental treatment, smoking habits (never, former or current), dental attendance pattern, frequency of intake of sugary food (specifically, chocolate, candy, ice cream, cakes, cookies, jam, marmalade, chocolate spread, and yoghurt) and/or drinks (specifically, hot drinks, soda, sports drink, and juice), and frequency of tooth brushing. The participants’ country of birth was dichotomised into ‘western’ (Nordic countries, Western Europe, North America, and Australia) and ‘non-western’ (the rest of the world), and the level of education was dichotomised into ‘higher education’ (university/college) and ‘basic education’ (high school, elementary school, or lower). Financial capacity related to affording dental treatment was assessed by the question “Have you experienced that dental treatment was affected by, or had to be postponed, due to the cost?” (yes or no). Furthermore, dental attendance pattern was dichotomised into ‘regular’ (regular check-ups at least every second year) and ‘irregular’ (occasionally, only emergency visits, or never). Frequency of sugary intake was dichotomised into ‘twice a week or less’ (seldom/never, 1–2 times per month, 1–2 times per week), and ‘several times a week/daily’ (3–4 times per week, daily, several times daily) [33]. Frequency of tooth brushing was dichotomised into ‘twice daily or more’ (more than once a day), and ‘less than twice daily’ (never, not every day, once a day).

### Clinical and radiographic examinations

The clinical and radiographic examinations were performed by two calibrated dentists (MTD and ATTS). Both dentists were present during all the examinations and reached a joint decision in cases of doubt related to caries registrations. For calibration, seven of the patient examinations (840 tooth surfaces) were performed by both examiners. The kappa value was 0.95 (95% CI: 0.94–0.96) for inter-examiner agreement on decayed, missing and filled surfaces, indicating almost perfect agreement.

Participants were instructed to refrain from eating, drinking, and smoking for at least 1 h before the clinical examination. Prior to the clinical examination, unstimulated whole saliva (UWS) was collected using a standardised protocol. Participants were instructed to sit relaxed and swallow any saliva in their mouth. The participants were asked to avoid swallowing saliva during the collection time, and they were instructed to spit into the test cup before swallowing, if they felt the urge to swallow. After 5 min, the participants were asked to spit any remaining saliva into the test cup. Pathologically low salivary secretion rate, hyposalivation, was defined as secretion rate of ≤ 0.1 ml/min for UWS [34].

Two bitewing radiographs were then taken, using an intraoral imaging unit with a rectangular 30.5 cm collimator (MINIRAY, SOREDEX, PaloDEx Group Oy, Tuusula, Finland). Additional bitewing radiographs were taken if the distal surface of the maxillary canines or any posterior approximal surface were not visible.

All participants then underwent a thorough clinical examination in a dental chair. Primary- and secondary coronal and root caries lesions, and dental restorations were recorded at the level of the tooth surface aided by the radiographic examinations. A five-grade scale was used to grade the coronal primary caries lesions, according to the diagnostic criteria described by Amarante and co-workers and used in several Norwegian epidemiological studies [4, 13, 19, 35]. Coronal primary caries lesions in enamel were registered as either grades 1 (outer half of the enamel) or 2 (inner half of the enamel), and dentine caries lesions as grades 3, 4 or 5 (outer, middle and inner third of the dentine). Secondary caries lesions were recorded without grading and were all classified as dentine caries. Root caries lesions were graded according to the International Caries Detection and Assessment System (ICDAS) for root caries [36]. Root surfaces were recorded as either (1) sound root surface without discolouration, (2) clearly demarcated discoloured area on the root surface but with no cavitation (loss of anatomical contour < 0.5 mm), and (3) root surface with discolouration and cavitation (loss of anatomical contour ≥ 0.5 mm).

Caries experience was quantified using the DMF-index (decayed, missing, filled) in accordance with the criteria by the World Health Organization [37], combining the recordings from both the crown and root surfaces as follows: The D-component included coronal dentine caries lesions, root caries lesions with ≥ 0.5 mm cavitation, and root remnants. Root remnants that were visible less than 2 mm above the gingival margin, were registered as four decayed surfaces (DS) for anterior teeth and five decayed surfaces (DS) for premolars and molars. If a tooth had a coronal caries lesion and/or a root caries lesion on the same surface, it was counted as having only one decayed surface when calculating the total DMFS score. The M-component comprised all missing teeth, regardless of the reason for the loss. Finally, the F-component comprised all restorations on coronal and root surfaces, including direct fillings and crowns, but not fixed dental prosthesis abutments. If a tooth had a coronal and/or root restoration on the same surface, it was counted as only having one filled surface (FS) when calculating the DMFS index. Third molars were not included in the registrations.

### Statistical analyses

Registrations were entered in the Oral Data Collector (ODC) sheet specifically designed for data entry in the present study. The ODC sheet was developed in Microsoft Excel 2016 (Microsoft Corporation, Redmond, Washington, USA), and processed using openpyxl 3.0.4 and pandas 1.1.0 in Python 3.8 (Python Software Foundation, https://www.python.org/) and was imported into STATA (Stata version 16.1; College Station, TX, USA) for statistical analysis. The data were securely stored and analysed within the Service for Sensitive Data (TSD) module (Centre for Information Technology Services, University of Oslo).

The outcome measures were mean number of remaining teeth, DMFT/S, and the presence of decayed teeth (DT > 0). The Mann-Whitney U test was used to determine significant differences in the distribution of caries experience related to the explanatory variables. For the further analyses, it was decided to focus on the parameter that reflected current disease, i.e. decayed teeth. Edentulous individuals (n = 2) were excluded from the bivariate and multivariable analyses where the presence of decayed teeth was the outcome. The Chi-square test was used to investigate the associations between the explanatory factors and the presence of decayed teeth. Furthermore, multivariable logistic regression analyses were used to further explore the associations between the explanatory factors and the presence of decayed teeth. Separate models for distant (sociodemographic variables, Table 1, Model 1), intermediate (behavioural variables, Table 1, model 2), and proximal biological influence (hyposalivation, Table 1, unadjusted odds ratio) are presented, accounting for hierarchical relationships between these independent variables. Finally, all explanatory variables were included in the same model (Table 1, Model 3). The results from the regression analyses are presented as unadjusted and adjusted odds ratios with a 95% confidence interval. The level of significance was set to *p* < 0.05.

**Table 1:** Logistic regression analysis: Presence of decayed teeth (DT > 0) in relation to selected explanatory variables among a sample of Norwegian older adults (n = 455)

			Model 1	Model 2	Model 3
N = 455		Unadjusted	Adjusted*	Adjusted*	Adjusted*
Explanatory variables		Odds ratio (95% CI)	Odds ratio (95% CI)	Odds ratio (95% CI)	Odds ratio (95% CI)
Sociodemographic variables					
Gender					
	Female	1	1		1
	Male	**2.1 (1.4-3.0)**	**2.0 (1.3–2.9)**		**1.8 (1.2–2.8)**
Country of birth					
	Western	1	1		1
	Non-western	**2.6 (1.3–4.9)**	1.8 (0.9–3.6)		1.3 (0.6–2.7)
Education level					
	Higher	1	1		1
	Basic	**2.1 (1.4–3.2)**	**1.9 (1.3-3.0)**		**1.9 (1.2–2.9)**
Financial capacity					
	Not limited	1	1		1
	Limited	**1.9 (1.1–3.2)**	1.5 (0.9–2.6)		1.2 (0.6–2.1)
Behavioural variables					
Smoking					
	Never	1		1	1
	Former	1.2 (0.8–1.9)		1.3 (0.9-2.0)	1.2 (0.8–1.9)
	Current	1.6 (0.9–3.1)		1.4 (0.7–2.7)	1.2 (0.6–2.4)
Dental attendance pattern					
	Regular	1		1	1
	Irregular	**3.8 (2.0–7.0)**		**3.2 (1.7–6.1)**	**2.2 (1.0-4.8)**
Toothbrushing					
	Twice daily or more	1		1	1
	Less than twice daily	**2.4 (1.4-4-0)**		**1.9 (1.1–3.3)**	1.6 (0.9–2.9)
Sugar intake					
	Twice a week or less	1		1	1
	More than twice a week	1.3 (0.9-2.0)		1.3 (0.9–1.9)	1.4 (0.9–2.1)
Biological variable					
Hyposalivation					
	No	1			1
	Yes	2.0 (1.0-3.9)			**2.1 (1.0-4.4)**

CI = confidence interval

Values shown in bold text differ significantly (p < 0.05: Logistic regression) from the reference category

*Adjusted for the variables in the same column

Pseudo R^2^ in Model 3 = 0.07

## Results

Of the 797 eligible participants who were reached by telephone after having received the invitation letter, 460 attended the clinical and radiographic examination (response rate of 58%). Three individuals were excluded from the analyses due to missing questionnaire data, and the final sample therefore comprised 457 individuals. The sample population was characterised by an even gender distribution (52% men, 48% women). They were predominantly born in a western country (91%) and had higher education (67%) (Table 2). 8% of the participants had hyposalivation. The participants had a mean number of 25 teeth (SD: 4, range: 0–28) and 94% had more than 20 remaining teeth. Two individuals were edentulous. Ninety-nine per cent of the participants had one or more exposed root surfaces, and the mean number of exposed root surfaces was 25 (SD: 18).

**Table 2:** Caries experience of the study participants in relation to selected explanatory variables among a sample of Norwegian older adults (n = 457)

			DMFT	MT	DT	FT	DMFS	DS	FS
Characteristics		N (%)	Mean (SD)	Mean (SD)	Mean (SD)	Mean (SD)	Mean (SD)	Mean (SD)	Mean (SD)
All		457 (100)	19.4 (4.7)	2.5 (3.9)	0.8 (1.6)	16.1 (5.4)	61.6 (20.2)	1.3 (3.7)	48.2 (20.8)
Sociodemographic variables									
Gender									
	Male	236 (52)	19.3 (5.2)	2.9 (4.6)	1.1 (2.0)^a^	15.3 (5.9)^a^	61.6 (22.1)	1.9 (4.9)^a^	45.8 (22.7)^a^
	Female	221 (48)	19.5 (4.2)	2.1 (2.8)	0.5 (1.1)^a^	16.8 (4.7)^a^	61.7 (18.0)	0.7 (1.5)^a^	50.8 (18.2)^a^
Country of birth									
	Western	414 (91)	19.7 (4.2)^a^	2.1 (2.9)^a^	0.7 (1.6)^a^	16.9 (4.6)^a^	62.5 (18.3)^a^	1.2 (3.8)^a^	51.3 (18.6)^a^
	Non-western	43 (9)	16.8 (7.8)^a^	6.9 (7.5)^a^	1.5 (2.0)^a^	8.4 (6.2)^a^	53.6 (32.6)^a^	2.4 (3.1)^a^	18.2 (17.0)^a^
Education level									
	Basic	152 (33)	19.6 (5.2)	3.6 (4.8)^a^	1.1 (2.0)^a^	14.9 (5.9)^a^	62.9 (22.0)	2.0 (5.2)^a^	43.3 (21.6)^a^
	Higher	305 (67)	19.3 (4.5)	1.9 (3.2)^a^	0.7 (1.4)^a^	16.7 (5.0)^a^	61.0 (19.3)	0.9 (2.7)^a^	50.6 (19.9)^a^
Financial capacity									
	Limited	73 (16)	19.4 (5.8)	3.6 (4.2)^a^	1.2 (2.2)^a^	14.6 (6.6)	61.0 (22.7)	2.5 (6.8)^a^	41.0 (23.7)^a^
	Not limited	384 (84)	19.4 (4.5)	2.3 (3.7)^a^	0.7 (1.5)^a^	16.3 (5.1)	61.8 (19.8)	1.0 (2.7)^a^	49.6 (20.0)^a^
Behavioural variables									
Smoking									
	Never	197 (43)	18.2 (4.9)^ab^	1.7 (2.5)^ab^	0.8 (1.6)	15.7 (5.4)	56.5 (19.3)^ab^	1.1 (2.7)	47.1 (20.1)
	Former	210 (46)	20.3 (4.4)^a^	2.6 (3.5)^a^	0.8 (1.4)	16.9 (5.0)^a^	64.7 (18.6)^a^	1.0 (2.6)	50.7 (20.6)^a^
	Current	50 (11)	20.5 (4.5)^b^	5.1 (7.1)^a^	1.1 (2.6)	14.3 (6.2)^a^	70.0 (25.3)^a^	2.6 (8.2)	42.2 (23.1)^a^
Dental attendance pattern									
	Irregular	52 (11)	18.2 (7.3)	6.6 (7.8)^a^	2.0 (2.7)^a^	9.6 (6.8)^a^	61.7 (32.6)	5.1 (9.2)^a^	25.4 (22.7)^a^
	Regular	405 (89)	19.5 (4.3)	2.0 (2.6)^a^	0.7 (1.4)^a^	16.9 (4.5)^a^	61.6 (18.1)	0.8 (1.7)^a^	51.1 (18.6)^a^
Toothbrushing									
	Twice daily or more	386 (84)	19.5 (4.4)	2.2 (3.3)	0.7 (1.5)^a^	16.5 (5.0)^a^	61.7 (18.9)	1.1 (3.3)^a^	49.9 (19.3)^a^
	Less than twice daily	71 (16)	18.9 (6.1)	4.0 (5.9)	1.3 (2.0)^a^	13.6 (6.7)^a^	60.9 (26.6)	2.4 (5.2)^a^	39.2 (26.0)^a^
Sugar intake									
	Twice a week or less	239 (52)	19.4 (4.4)	2.3 (2.7)	0.7 (1.7)	16.4 (5.0)	61.9 (18.4)	1.2 (4.0)	49.6 (20.0)
	More than twice a week	218 (48)	19.3 (5.1)	2.8 (4.8)	0.9 (1.6)	15.7 (5.7)	61.4 (22.1)	1.4 (3.4)	46.7 (21.5)
Biological variable									
Hyposalivation									
	No	421 (92)	19.4 (4.6)	2.5 (3.9)^a^	0.7 (1.4)^a^	16.2 (5.3)	61.3 (19.7)	1.1 (2.5)^a^	48.2 (20.4)
	Yes	36 (8)	19.0 (5.9)	2.9 (2.5)^a^	1.8 (3.2)^a^	14.3 (6.4)	66.0 (25.3)	3.7 (9.8)^a^	48.0 (25.1)

Letters indicate a statistically significant difference between groups of the same letter within the same variable (p < 0.05: Mann-Whitney U test)

### Caries experience (DMFT/S)

The participant’s caries experience in relation to selected explanatory variables is presented as mean values in Table 2 and as median values in a supplementary table (Table S1). The mean DMFT for the study sample was 19.4 (SD: 4.7). The mean DT was 0.8 (SD: 1.6) and the mean MT was 2.5 (SD: 3.9). 37% of the individuals had at least one decayed tooth (DT > 0). A higher DT/DS was significantly associated with male gender, non-western origin, basic level of education, irregular dental attendance, limited financial capacity, toothbrushing less frequently than twice daily, and hyposalivation. The filled teeth (FT) component made up the largest part of the overall mean DMFT score, with an average of 16.1 filled teeth per person (SD: 5.4). A higher FT/FS was significantly associated with female gender, western origin, higher level of education, not having limited financial capacity, being a former smoker (compared to current smokers) and regular dental attendance.

### Prevalence of coronal and root caries

The prevalence of enamel caries was 35%, and the prevalence of the other caries classifications was as follows: primary coronal dentine caries 12%, secondary coronal caries in dentine 33%, root caries without cavitation 17%, and root caries with cavitation 7% (Table 3). Thirteen participants (3%) had one or more root remnants (range: 1–9).

**Table 3:** Distribution of participants according to the presence of coronal and root caries lesions among a sample of Norwegian older adults (n = 457)

N = 457 No. of lesions	Enamel caries (%)	Coronal dentine caries (%)	Secondary caries (%)	Root caries without cavitation* (%)	Root caries with cavitation** (%)
0	65	88	67	83	93
1	19	8	19	11	5
2	7.4	2.6	5.9	3.3	1.5
3	3.9	0.4	3.7	0.9	0
4	1.8	0.2	1.8	1.1	0
5	1.8	0.4	0.7	0.4	0
6	0.4	0	0.2	0.2	0
7	0.2	0	0.4	0.2	0.2
8	0	0	0.9	0.2	0
12	0	0	0.2	0	0
13	0.2	0	0	0	0
20	0	0	0	0	0.2

* < 0.5 mm cavitation

** ≥ 0.5 mm cavitation

### Risk indicators for decayed teeth

The prevalence of decayed teeth (DT > 0) was 37%, and its distribution according to sociodemographic, behavioural, and biological factors is presented in Table 4. Multivariable logistic regression analyses showed that male gender and basic level of education were significant risk indicators of having decayed teeth in the model including only the sociodemographic variables (Table 1, model 1), while irregular dental attendance and toothbrushing less than twice daily were significant risk indicators in the model including only the behavioural variables (Table 1, model 2). When the sociodemographic, behavioural, and biological variables were all included in the same model (Table 1, model 3), having male gender, a basic level of education, irregular dental attendance and hyposalivation were significantly related to having one or more decayed teeth.

**Table 4:** Bivariate associations between the prevalence of decayed teeth (DT > 0) and selected explanatory variables among a sample of Norwegian older adults (n = 455)

N = 455		DT > 0 n (%)	
All		171 (38*)	
Sociodemographic variables			
Gender			
	Male	107 (46)^a^	
	Female	64 (29)^a^	
Country of birth			
	Western	147 (36)^a^	
	Non-western	24 (59)^a^	
Education level			
	Basic	75 (50)^a^	
	Higher	96 (32)^a^	
Financial capacity			
	Limited	37 (51)^a^	
	Not limited	134 (35)^a^	
Behavioural variables			
Smoking			
	Never	67 (34)	
	Former	82 (39)	
	Current	22 (46)	
Dental attendance pattern			
	Irregular	33 (66)^a^	
	Regular	138 (34)^a^	
Toothbrushing			
	Twice daily or more	132 (34)^a^	
	Less than twice daily	39 (56)^a^	
Sugar intake			
	Twice a week or less	82 (34)	
	More than twice a week	89 (41)	
Biological variable			
Hyposalivation			
	No	152 (36)^a^	
	Yes	19 (53)^a^	

Letters in superscript indicate a statistically significant difference between groups with the same letter within the same variable (p < 0.05: Chi-square test)

*37% if the edentulous (n = 2) are included

## Discussion

This paper describes the caries experience in a random sample of 65-year-old urban Norwegians. In addition, associations between having decayed teeth and sociodemographic- and behavioural variables as well as hyposalivation were explored. To our knowledge, few studies have explored the association between caries prevalence and clinically measured hyposalivation in a study sample from the general population.

### Caries experience

A recent review has summarized the caries experience in European citizens aged 65–74 years that has been reported in studies in the period 1996–2016 [38]. The mean DMFT scores varied among 20 countries between 14.6 and 25.8. The authors observed a trend towards lower MT scores and higher FT scores during this period. Regardless, the MT component represented the largest component of the DMFT score in almost all countries in this age group, unlike in the present study, where the FT component was the most predominant component. Restored tooth surfaces have a higher risk of developing caries than sound tooth surfaces [39]. Hence, this trend towards the retention of more teeth later in life (lower MT), and more restorative therapy (higher FT) [38], is likely to increase the need for maintenance of restored teeth in older adults as this cohort ages. The high retention of teeth in the present young-elderly in Norway compared to many other European countries, may be partly attributed to the good access to, and high utilization of dental services in Norway, where 10 years ago 80% of adults were reported to have visited the dentist within the last year [40].

The caries experience in the present study population in Oslo (DMFT 19.4) was slightly lower than in similar studies from the central (DMFT 21.0) and northern (DMFT 21.5–22.5) parts of Norway [4, 13, 19]. Although the mean number of decayed teeth in the present study (DT = 0.8) was within the range reported in the other Norwegian studies (0.2–1.5) [4, 13, 19], in the present study the mean number of missing teeth was lower (MT = 2.5 versus 6.1–9.2), and the mean number of filled teeth was higher (FT = 16.1 versus 12.4–15.2). The lower level of caries experience in the city of Oslo compared to the other Norwegian studies may be partly explained by the fact that those study samples were of somewhat older age groups (65–74 years). Furthermore, the other study samples were from more rural areas that are known to have higher levels of dental caries [13]. Moreover, there is better access to dental care in the Oslo area compared to in the central and northern parts of Norway where patients may need to travel some distance to clinics [27], which may have contributed to the lower numbers of MT and higher numbers of FT in Oslo. A study from Northern Norway showed that 32% of adults 30–59 years and 26% of adults ≥ 60 years used dental health services at least every second year [41]. These are lower proportions compared to the corresponding figure in the present study on 65-year-olds in Oslo (66%).

In comparison, the mean DFT score in the present study (16.8) was very similar to that reported in 70-year-olds in Sweden (16.6) [2]. However, the mean DMFT scores among comparable age groups in Denmark (age 65–74 y: mean DMFT = 23.1) [3] and Finland (age 65–74 y: mean DMFT = 25.6) [42] were higher than in the present study. The Danish and Finnish studies were conducted more than ten years ago, and in older age groups, and the DMFT scores in these countries may therefore be somewhat lower now.

A recent report from the United States (US) showed a mean DMFT score of 15.9 among dentate 65-74-year-olds in the resident, civilian, noninstitutionalized US population. The mean DT (0.3) and FT (9.9) were lower than in the present study, and the mean MT was higher (5.6) [43]. The prevalence of edentulism in the US study was 13% compared to 0.4% in the present study, so the inclusion of missing teeth in the edentulous population would have increased the mean DMFT score estimate to 17.4. In general, more tooth extractions and less restorative caries treatment in the US compared to the general practice in Norway, may be partly explained by less affordable dental health services in the US [44].

### Prevalence of coronal and root caries

In the present study, both enamel and dentine caries lesions were recorded, but only caries lesions that had reached dentine were included in the DMFT score. Enamel caries- and root caries lesions without cavitation are therefore reported separately.

More than one of three individuals in our study had untreated dentine caries (DT > 0), which is in line with recent data from Northern Norway (35–39%) [13, 19]. During the last decade, apart from the Norwegian studies, few European studies have reported caries prevalence in this age group from a general population sample. A caries prevalence of 49% was recently reported in Polish 65-74-year-olds [45], indicating a higher caries treatment need in this age group than in Norway. Although similar figures from the US showed a caries prevalence of 15% among dentate 65–74 year-olds [43], root caries was not included in these figures. In the present study, teeth with secondary caries lesions represented the largest proportion of decayed teeth. This was not surprising considering the high proportion of filled tooth surfaces among the participants.

The overall prevalence of root caries (20%) in the present study was higher than reported in previous Norwegian studies (14–15%) [19, 20], but lower than in studies from Turkey (28%) [46] and Greece (38%) [47], from similar age groups. In the present study, exposed root surfaces were frequent, and more than one-third of participants had unstable periodontitis [48]. Gingival retractions and subsequently exposed root surfaces are preconditions for root caries and have been reported as a root caries risk indicator [49, 50]. Therefore, the periodontal status in the present study sample may indicate that one can expect an increased prevalence of root caries as this population ages further.

### Risk indicators for decayed teeth

In the present study, 8% of the participants had hyposalivation with respect to unstimulated whole saliva secretion. Saliva protects against caries as it is a reservoir of tooth minerals, clears food and helps to neutralise plaque pH after eating, and contains antimicrobial components [51]. In line with a previous study [52], the present study showed an association between hyposalivation and prevalence of decayed teeth (Table 1, model 3). Furthermore, the use of multiple medications, which is increasingly common among older adults [53], is associated with hyposalivation [31, 54]. Therefore, hyposalivation and accompanying problems, such as a higher risk of developing root caries [49], may be expected to increase in this population as they get older.

Previous studies have reported associations between the presence of dental caries and male gender [4, 19, 55], lower level of education [13, 56], irregular dental attendance [13, 57], and toothbrushing less than twice daily [13]. These findings were confirmed in the present study. Logistic regression analyses showed that having male gender and a basic level of education were significantly associated with having decayed teeth, after adjustment for the other sociodemographic variables (Table 1, model 1). Furthermore, irregular dental attendance and toothbrushing less than twice daily were significantly associated with having decayed teeth after adjustment for the other behavioural variables (Table 1, model 2). The association between the sociodemographic factors and caries may be partly explained by behavioural patterns [18, 58]. In the present study, males, and individuals with only a basic level of education visited the dentist less regularly, and brushed their teeth less often than their counterparts, which may have contributed to the higher prevalence of decayed teeth in these groups. Of those who reported brushing their teeth at least twice daily in the present study (n = 386), the majority reported using fluoridated toothpaste on a daily basis (89%). It has been suggested that biological and behavioural factors serve as stronger predictors of caries than sociodemographic factors because they have a more direct effect on the development of the disease [22]. This concurs with the present study where irregular dental attendance and hyposalivation were slightly stronger risk indicators for having decayed teeth than the sociodemographic variables (Table 1, model 3). Regular dental attendance may lead to a more successful prevention of caries and also early intervention with restorative treatment when necessary, thus reducing the caries prevalence in that group.

### Limitations of the study

In line with many studies that are performed in specific population groups, the response rate of 58% reflected a considerable proportion of non-attenders. This figure is comparable to previous studies with similar recruitment procedures (51–64%) [59, 60], and due to restrictions from the ethics committee, we were not permitted to ask non-attenders why they declined to participate. Therefore, to explore potential selection bias, the gender distribution and education level of the study sample were compared with the reported data on 65-year-olds living in Oslo, retrieved from Statistics Norway. Although gender distribution was similar, the proportion of participants with a higher level of education in the present study sample was higher than in the source population. Thus, selection bias may have affected the caries-related prevalence estimates. Given that the DT- and MT components were higher in those with only basic level of education, the actual prevalence of decayed and missing teeth in the present study may have been underestimated.

## Conclusions

The present study indicates that while the young-elderly population in Oslo have few decayed and missing teeth, they have many restored teeth. Coronal secondary dentine caries lesions comprised the largest proportion of the total burden of decay. Irregular dental attendance and hyposalivation were the strongest risk indicators for having decayed teeth in this population. Furthermore, exposed root surfaces that are at risk of developing root caries lesions, were very common. Based on the present results, it will be important to ensure access to regular dental care and to increase the emphasis on caries preventive measures, especially for individuals with hyposalivation, in this age group.

### Electronic supplementary material

Below is the link to the electronic supplementary material.

Supplementary Material 1

## Data Availability

The data that support the findings of the present study are available on request from the corresponding author. The data are not publicly available due to privacy or ethical restrictions.

## Abbreviations

DMFT/S: Decayed, missing, filled teeth/surfaces
UWS: Unstimulated whole saliva
CI: Confidence interval
SD: Standard deviation

## References

[1] Ettinger RL, Marchini L (2020). Cohort differences among aging populations: an update. J Am Dent Assoc.

[2] Norderyd O, Koch G, Papias A, Kohler AA, Helkimo AN, Brahm CO (2015). Oral health of individuals aged 3–80 years in Jonkoping, Sweden during 40 years (1973–2013). II. Review of clinical and radiographic findings. Swed Dent J.

[3] Kongstad J, Ekstrand K, Qvist V, Christensen LB, Cortsen B, Grønbaek M (2013). Findings from the oral health study of the danish health examination Survey 2007–2008. Acta Odontol Scand.

[4] Rødseth SC, Høvik H, Schuller AA, Bjertness E, Skudutyte-Rysstad R. Dental caries in a norwegian adult population, the HUNT4 oral health study; prevalence, distribution and 45-year trends. Acta Odontol Scand. 2022:1–9. 10.1080/00016357.2022.2117735 36150007

[5] Douglass CW, Sheets CG (2000). Patients’ expectations for oral health care in the 21st century. J Am Dent Assoc.

[6] Marengoni A, Angleman S, Melis R, Mangialasche F, Karp A, Garmen A (2011). Aging with multimorbidity: a systematic review of the literature. Ageing Res Rev.

[7] Ahmad MS, Bhayat A, Zafar MS, Al-Samadani KH. The impact of Hyposalivation on Quality of Life (QoL) and oral health in the Aging Population of Al Madinah Al Munawarrah. Int J Environ Res Public Health. 2017;14(4). 10.3390/ijerph14040445 PMC5409645 28425972

[8] Saunders RH, Meyerowitz C (2005). Dental caries in older adults. Dent Clin North Am.

[9] Yu LX, Wang X, Feng XP, Tai BJ, Hu Y, Wang B (2021). The relationship between different types of caries and periodontal disease severity in middle-aged and elderly people: findings from the 4th national oral health survey of China. BMC Oral Health.

[10] Fure S, Zickert I (1997). Incidence of tooth loss and dental caries in 60-, 70- and 80-year-old swedish individuals. Community Dent Oral Epidemiol.

[11] Machiulskiene V, Campus G, Carvalho JC, Dige I, Ekstrand KR, Jablonski-Momeni A (2020). Terminology of Dental Caries and Dental Caries Management: Consensus Report of a Workshop Organized by ORCA and Cariology Research Group of IADR. Caries Res.

[12] Jensen O, Gabre P, Sköld UM, Birkhed D (2012). Is the use of fluoride toothpaste optimal? Knowledge, attitudes and behaviour concerning fluoride toothpaste and toothbrushing in different age groups in Sweden. Community Dent Oral Epidemiol.

[13] Oscarson N, Espelid I, Jönsson B (2017). Is caries equally distributed in adults? A population-based cross-sectional study in Norway - the TOHNN-study. Acta Odontol Scand.

[14] Peres MA, Sheiham A, Liu P, Demarco FF, Silva AE, Assunção MC (2016). Sugar Consumption and Changes in Dental Caries from Childhood to Adolescence. J Dent Res.

[15] Edman K, Öhrn K, Nordström B, Holmlund A (2016). Prevalence of dental caries and influencing factors, time trends over a 30-year period in an adult population. Epidemiological studies between 1983 and 2013 in the county of Dalarna, Sweden. Acta Odontol Scand.

[16] Skudutyte-Rysstad R, Eriksen HM (2007). Changes in caries experience among 35-year-old Oslo citizens, 1973–2003. Acta Odontol Scand.

[17] Costa SM, Martins CC, Bonfim Mde L, Zina LG, Paiva SM, Pordeus IA (2012). A systematic review of socioeconomic indicators and dental caries in adults. Int J Environ Res Public Health.

[18] Schwendicke F, Dörfer CE, Schlattmann P, Foster Page L, Thomson WM, Paris S (2015). Socioeconomic inequality and caries: a systematic review and meta-analysis. J Dent Res.

[19] Mulic A, Tveit AB, Stenhagen KR, Oscarson N, Staxrud F, Jönsson B (2020). The frequency of enamel and dentin caries lesions among elderly Norwegians. Acta Odontol Scand.

[20] Henriksen BM, Ambjørnsen E, Axéll T (2004). Dental caries among the elderly in Norway. Acta Odontol Scand.

[21] Beenackers MA, Vermaire JH, van Dommelen P, Schuller AA (2021). Experiencing Financial strain and clinically assessed Caries experience in dentate adults aged 25–44 years: an exploration of potential pathways. Caries Res.

[22] Aleksejūnienė J, Holst D, Brukienė V (2009). Dental caries risk studies revisited: causal approaches needed for future inquiries. Int J Environ Res Public Health.

[23] Mullen J (2005). History of water fluoridation. Br Dent J.

[24] Vidnes-Kopperud S, Tveit AB, Espelid I (2011). Changes in the treatment concept for approximal caries from 1983 to 2009 in Norway. Caries Res.

[25] Tveit AB, Espelid I, Skodje F (1999). Restorative treatment decisions on approximal caries in Norway. Int Dent J.

[26] Grytten J, Holst D, Skau I (2009). Incentives and remuneration systems in dental services. Int J Health Care Finance Econ.

[27] Grytten J, Skau I (2009). Specialization and competition in dental health services. Health Econ.

[28] United Nations (2020). World Population Ageing 2019. Department of Economic and Social Affairs.

[29] OECD. Organisation for Economic Co-operation and Development (OECD) [Internet]. Elderly population. [cited 2022 Oct 01]. Available from: https://data.oecd.org/pop/elderly-population.htm 2022 [.

[30] Chan AKY, Tamrakar M, Jiang CM, Lo ECM, Leung KCM, Chu CH. A systematic review on Caries Status of older adults. Int J Environ Res Public Health. 2021;18(20). 10.3390/ijerph182010662 PMC8535396 34682414

[31] Diep MT, Jensen JL, Skudutyte-Rysstad R, Young A, Sødal ATT, Petrovski BÉ (2021). Xerostomia and hyposalivation among a 65-yr-old population living in Oslo, Norway. Eur J Oral Sci.

[32] von Elm E, Altman DG, Egger M, Pocock SJ, Gøtzsche PC, Vandenbroucke JP (2008). The strengthening the reporting of Observational Studies in Epidemiology (STROBE) statement: guidelines for reporting observational studies. J Clin Epidemiol.

[33] The Norwegian Directorate of Health. Helsedirektoratet [Internet]. 10. Unngå mat og drikke med mye sukker til hverdags. [cited 2023 Jun 06]. Available from: https://www.helsedirektoratet.no/faglige-rad/kostradene-og-naeringsstoffer/kostrad-for-befolkningen/unnga-mat-og-drikke-med-mye-sukker-til-hverdags 2016 [.

[34] Vitali C, Bombardieri S, Jonsson R, Moutsopoulos HM, Alexander EL, Carsons SE (2002). Classification criteria for Sjögren’s syndrome: a revised version of the european criteria proposed by the american-european Consensus Group. Ann Rheum Dis.

[35] Amarante E, Raadal M, Espelid I (1998). Impact of diagnostic criteria on the prevalence of dental caries in norwegian children aged 5, 12 and 18 years. Community Dent Oral Epidemiol.

[36] Gugnani N, Pandit IK, Srivastava N, Gupta M, Sharma M (2011). International Caries Detection and Assessment System (ICDAS): a New Concept. Int J Clin Pediatr Dent.

[37] Petersen P, Baez R. World Health Organization. Section 1: Basic principles of clinical oral health surveys. Oral health surveys: basic methods. 5th ed. World Health Organization; 2013.

[38] Carvalho JC, Schiffner U (2019). Dental Caries in european adults and senior Citizens 1996–2016: ORCA Saturday Afternoon Symposium in Greifswald, Germany - Part II. Caries Res.

[39] Kirkevang LL, Væth M, Wenzel A (2011). Incidence of caries lesions in approximal surfaces: a radiographic study of a general adult danish population. Caries Res.

[40] Grytten J, Holst D, Skau I (2012). Demand for and utilization of dental services according to household income in the adult population in Norway. Community Dent Oral Epidemiol.

[41] Hadler-Olsen E, Jönsson B (2021). Oral health and use of dental services in different stages of adulthood in Norway: a cross sectional study. BMC Oral Health.

[42] Suominen-Taipale L, Nordblad A, Vehkalahti M, Aromaa A (2008). Oral health in the Finnish Adult Population. Health 2000 Survey.

[43] Centers for Disease Control and Prevention. Oral Health Surveillance Report (2019). Trends in Dental Caries and Sealants, tooth Retention, and Edentulism, United States, 1999–2004 to 2011–2016.

[44] Vujicic M, Buchmueller T, Klein R (2016). Dental Care presents the highest level of Financial Barriers, compared to other types of Health Care Services. Health Aff (Millwood).

[45] Głowacka B, Konopka T (2019). Needs for gerodontological treatment in the elderly living in Lower Silesia. Dent Med Probl.

[46] Gökalp SG, Doğan BG, Tekçiçek MT, Berberoğlu A, Unlüer S (2010). National survey of oral health status of children and adults in Turkey. Community Dent Health.

[47] Mamai-Homata E, Topitsoglou V, Oulis C, Margaritis V, Polychronopoulou A (2012). Risk indicators of coronal and root caries in greek middle aged adults and senior citizens. BMC Public Health.

[48] Sødal ATT, Hove LH, Diep MT, Skudutyte-Rysstad R, Koldsland OC (2022). Periodontal conditions in a 65-year-old population and prevalence of periodontitis according to three different bone level thresholds. BMC Oral Health.

[49] Hayes M, Da Mata C, Cole M, McKenna G, Burke F, Allen PF (2016). Risk indicators associated with root caries in independently living older adults. J Dent.

[50] Zhang J, Leung KCM, Chu CH, Lo ECM (2020). Risk indicators for root caries in older adults using long-term social care facilities in Hong Kong. Community Dent Oral Epidemiol.

[51] Whelton H. Introduction: the anatomy and physioloy of salivary glands. In: Edgar M, Dawes, C., O’Mullane, D., editor. Saliva and oral health. 4th ed: Stephen Hancocks Limited 2012. p. 1–16.

[52] Flink H, Tegelberg A, Sörensen S (2000). Hyposalivation and iron stores among individuals with and without active dental caries. Acta Odontol Scand.

[53] Wastesson JW, Morin L, Tan ECK, Johnell K (2018). An update on the clinical consequences of polypharmacy in older adults: a narrative review. Expert Opin Drug Saf.

[54] Smidt D, Torpet LA, Nauntofte B, Heegaard KM, Pedersen AML (2011). Associations between oral and ocular dryness, labial and whole salivary flow rates, systemic diseases and medications in a sample of older people. Community Dent Oral Epidemiol.

[55] Suominen AL, Varsio S, Helminen S, Nordblad A, Lahti S, Knuuttila M. Dental and periodontal health in finnish adults in 2000 and 2011. Acta Odontol Scand. 2018:1–9. 10.1080/00016357.2018.1451653 29546776

[56] Lambert M, De Reu G, De Visschere L, Declerck D, Bottenberg P, Vanobbergen J (2018). Social gradient in caries experience of belgian adults 2010. Community Dent Health.

[57] Bongo AS, Brustad M, Jönsson B (2021). Caries experience among adults in core Sami areas of Northern Norway. Community Dent Oral Epidemiol.

[58] Singh A, Rouxel P, Watt RG, Tsakos G (2013). Social inequalities in clustering of oral health related behaviors in a national sample of british adults. Prev Med.

[59] Kirkevang LL, Horsted-Bindslev P, Ørstavik D, Wenzel A (2001). Frequency and distribution of endodontically treated teeth and apical periodontitis in an urban danish population. Int Endod J.

[60] Skudutyte-Rysstad R, Eriksen HM (2006). Endodontic status amongst 35-year-old Oslo citizens and changes over a 30-year period. Int Endod J.

