# Supplementary material for: Caries experience and risk indicators of having decayed teeth among 65-year-olds in Oslo, Norway: a cross-sectional study
Source: BMC Oral Health. 2023 Oct 7;23:726. doi: 10.1186/s12903-023-03432-x (PMC10559471; doi:10.1186/s12903-023-03432-x)
Supplement: Supplementary file 1 — Supplementary Material 1 [file 12903_2023_3432_MOESM1_ESM.docx]

| **Table S1.** Caries experience of the study participants in relation to selected explanatory variables among a sample of Norwegian older adults (n=457) | | | | | | | |  |  |
| --- | --- | --- | --- | --- | --- | --- | --- | --- | --- |
|  |  |  | DMFT | MT | DT | FT | DMFS | DS | FS |
| Characteristics | | N (%) | Median (IQR) | Median (IQR) | Median (IQR) | Median (IQR) | Median (IQR) | Median (IQR) | Median (IQR) |
|  |  |  |  |  |  |  |  |  |  |
| All |  | 457 (100) | 20 (16-22) | 1 (0-4) | 0 (0-1) | 17 (13-20) | 62 (50-74) | 0 (0-1) | 50 (34-63) |
|  |  |  |  |  |  |  |  |  |  |
| Sociodemographic variables | |  |  |  |  |  |  |  |  |
|  |  |  |  |  |  |  |  |  |  |
| Gender | |  |  |  |  |  |  |  |  |
|  | Male | 236 (52) | 20 (16-23) | 1 (0-4) | 0 (0-1)^a^ | 16 (12-20)^a^ | 62 (49.5-75) | 0 (0-2)^a^ | 50 (29.5-61)^a^ |
|  | Female | 221 (48) | 20 (17-22) | 1 (0-4) | 0 (0-1)^a^ | 17 (14-20)^a^ | 61 (50-73) | 0 (0-1)^a^ | 52 (38-64)^a^ |
| Country of birth | |  |  |  |  |  |  |  |  |
|  | Western | 414 (91) | 20 (17-22)^a^ | 1 (0-3)^a^ | 0 (0-1)^a^ | 17 (14-20)^a^ | 62.5 (51-75)^a^ | 0 (0-1)^a^ | 53 (38-64)^a^ |
|  | Non-western | 43 (9) | 18 (9-21)^a^ | 4 (2-11)^a^ | 0 (0-2)^a^ | 7 (3-14)^a^ | 54 (32-69)^a^ | 0 (0-4)^a^ | 13 (4-29)^a^ |
| Education | |  |  |  |  |  |  |  |  |
|  | Basic | 152 (33) | 20 (17-23) | 2 (1-4)^a^ | 0 (0-1)^a^ | 16 (11-19)^a^ | 64 (51-77) | 0.5 (0-2)^a^ | 45 (29.5-60)^a^ |
|  | Higher | 305 (67) | 20 (16-22) | 1 (1-3)^a^ | 0 (0-1)^a^ | 17 (14-20)^a^ | 60 (49-73) | 0 (0-1)^a^ | 53 (37-64)^a^ |
| Financial capacity | |  |  |  |  |  |  |  |  |
|  | Limited | 73 (16) | 20 (17-23) | 2 (1-5))^a^ | 0 (0-1)^a^ | 16 (9-20) | 62 (49-73) | 0 (0-2)^a^ | 43 (23-60)^a^ |
|  | Not limited | 384 (84) | 20 (16-22) | 1 (0-3)^a^ | 0 (0-1)^a^ | 17 (14-20) | 62 (50-74.5) | 0 (0-1)^a^ | 51 (35-64)^a^ |
|  |  |  |  |  |  |  |  |  |  |
| Behavioural variables | |  |  |  |  |  |  |  |  |
|  |  |  |  |  |  |  |  |  |  |
| Smoking | |  |  |  |  |  |  |  |  |
|  | Never | 197 (43) | 19 (15-22)^ab^ | 1 (0-3)^ab^ | 0 (0-1) | 16 (13-20) | 58 (44-70)^ab^ | 0 (0-1) | 50 (33-61) |
|  | Former | 210 (46) | 21 (18-23)^a^ | 2 (0-4)^a^ | 0 (0-1) | 18 (14-20)^a^ | 65 (54-77)^a^ | 0 (0-1) | 53 (38-64)^a^ |
|  | Current | 50 (11) | 21 (18-23)^b^ | 2 (1-6)^a^ | 0 (0-1) | 15 (11-19)^a^ | 68 (53-84)^a^ | 0 (0-1) | 44 (29-61)^a^ |
| Dental visits | |  |  |  |  |  |  |  |  |
|  | Irregular | 52 (11) | 19.5 (13.5-24) | 3.5 (1.5-9)^a^ | 0 (0-3)^a^ | 9.5 (3-15)^a^ | 63 (38.5-78.5) | 2 (0-5.5)^a^ | 22 (5-40.5)^a^ |
|  | Regular | 405 (89) | 20 (17-22) | 1 (0-3)^a^ | 0 (0-1)^a^ | 17 (14-20)^a^ | 62 (51-74) | 0 (0-1)^a^ | 53 (39-64)^a^ |
| Toothbrushing | |  |  |  |  |  |  |  |  |
|  | Twice daily or more | 386 (84) | 20 (17-22) | 1 (0-4) | 0 (0-1)^a^ | 17 (14-20)^a^ | 62 (51-73) | 0 (0-1)^a^ | 52 (37-64)^a^ |
|  | Less than twice daily | 71 (16) | 20 (15-23) | 2 (0-5) | 0 (0-2)^a^ | 13 (9-19)^a^ | 62 (44-78) | 0 (0-3)^a^ | 35 (20-62)^a^ |
| Sugar intake | |  |  |  |  |  |  |  |  |
|  | Twice a week or less | 239 (52) | 20 (17-22) | 1 (1-4) | 0 (0-1) | 17 (14-20) | 62 (51-74) | 0 (0-1) | 51 (38-64) |
|  | More than twice a week | 218 (48) | 20 (16-23) | 1 (0-3) | 0 (0-1) | 16 (13-20) | 61 (47-74) | 0 (0-1) | 50 (32-61) |
|  |  |  |  |  |  |  |  |  |  |
| Biological variable | |  |  |  |  |  |  |  |  |
|  |  |  |  |  |  |  |  |  |  |
| Hyposalivation | |  |  |  |  |  |  |  |  |
|  | No | 421 (92) | 20 (16-22) | 1 (0-4)^a^ | 0 (0-1)^a^ | 17 (13-20) | 61 (50-73) | 0 (0-1)^a^ | 50 (34-63) |
|  | Yes | 36 (8) | 19.5 (16.5-23) | 2.5 (1-4)^a^ | 0 (0-2)^a^ | 15 (10-19) | 67.5 (50-79.5) | 1 (0-2.5)^a^ | 50.5 (32-62.5) |
|  |  |  |  |  |  |  |  |  |  |
| Letters indicate a statistically significant difference between groups of the same letter within the same variable (p < 0.05: Mann-Whitney U test) | | | | | | | | | |
